# *Ottelia fengshanensis*, a new bisexual species of *Ottelia* (Hydrocharitaceae) from southwestern China

**DOI:** 10.3897/phytokeys.135.38531

**Published:** 2019-10-30

**Authors:** Zhi-Zhong Li, Shuang Wu, Chun-Yu Zou, Yan Liu, Guang-Wan Hu, Samuli Lehtonen, Qing-Feng Wang, Jin-Ming Chen

**Affiliations:** 1 CAS Key Laboratory of Aquatic Botany and Watershed Ecology, Wuhan Botanical Garden, Chinese Academy of Sciences, Wuhan, CN-430074, China Wuhan Botanical Garden, Chinese Academy of Sciences Wuhan China; 2 University of Chinese Academy of Sciences, Beijing, CN-100049, China University of Chinese Academy of Sciences Beijing China; 3 Guangxi Association for Science and Technology, CN-530022, China Guangxi Association for Science and Technology Guangxi China; 4 Guangxi Institute of Botany, Chinese Academy of Sciences, Guilin, CN-541006, China Guangxi Institute of Botany, Chinese Academy of Sciences Guilin China; 5 Sino-Africa Joint Research Center, Chinese Academy of Sciences, Wuhan, CN-430074, China Sino-Africa Joint Research Center, Chinese Academy of Sciences Wuhan China; 6 Herbarium, Biodiversity Unit, University of Turku FI-20014 Turku, Finland University of Turku Turku Finland

**Keywords:** karst, bisexual flowers, molecular phylogeny

## Abstract

*Ottelia fengshanensis*, a new species (Hydrocharitaceae) from southwest China is here described and illustrated. Comparing its morphological features to putative close relatives *O. guanyangensis*, it has 3–4 flowers (vs. 2–5) each spathe, hexagonal-cylindric fruit, white styles (vs. yellow), green leaves (vs. dark green) and fruit tiny winged (vs. winged obviously). Molecular phylogenetic investigation of four DNA sequences (ITS, *rbc*L, *trn*K5’ intron and *trn*S-*trn*G) and the Poisson Tree Processes model for species delimitation (PTP) analysis, further resolves *O. fengshanensis* as a new species that is close to *O. guanyangensis* with distinct support.

## Introduction

*Ottelia*Persoon (1805:1) has about 22 species and is widely distributed in the tropical, subtropical and temperate regions. In comparison with other genera within the family Hydrocharitaceae, *Ottelia* is morphologically complex and variable, e.g. the leaf type of the genus is exceptionally erratic even within an individual depending on the developmental stage, as well as within the varieties or populations (Li et al. 2018). The flower sexuality varies within species and flowers can be either bisexual or unisexual. Southwestern China possesses complex terrain and various ecosystems and is a center of diversity for *Ottelia* species (Chen et al. 2017, Zhai et al. 2018). To date, six species and three varieties of *O. acuminata*Dandy (1934: 132) have been recorded from the area with narrowly endemic distribution in karst rivers or lakes. Among these, just three species, *O. alismoides*Persoon (1805: 273), *O. balansae*Dandy (1934: 137) and *O. guanyangensis* Z.Z. Li, Q.F. Wang & S. Wu (2018: 294) are bisexual and can only be found in specific karst regions, except for the widespread species *O. alismoides* (Cook et al. 1984, Cook and Urmi-Konig 1984, Li 1981).

**Figure 1.**
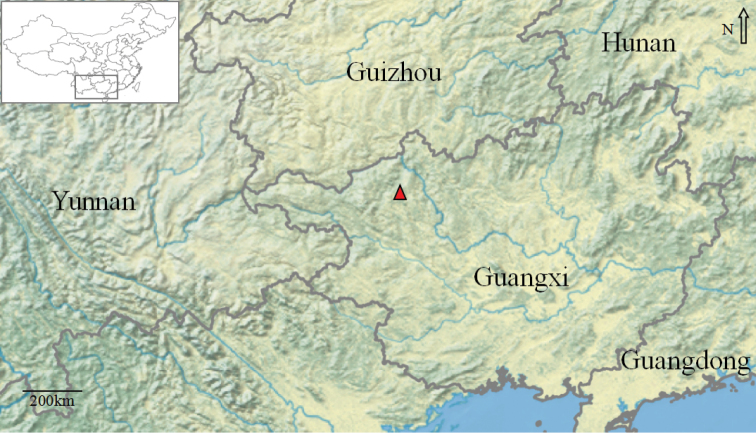
Distribution record of *Ottelia fengshanensis* Z.Z.Li, S.Wu & Q.F.Wang (red triangle) from Fengshan county, Guangxi province, China.

In 2017–2018, we found and reported a new bisexual species *O. guanyangensis* in Guilin City, China (Li et al. 2018). We deemed that there are some previously undetected potentially new *Ottelia* species in Guangxi province’s karst steams (Fig. 1). We made further aquatic plant investigations in Guangxi province, China, in 2018. From the Fengshan County, we found once again a species with bisexual flowers which generally appeared to be like *O. balansae*. Based on investigations of herbarium specimens in GXMG, HIB, IBSC, KUN and PE, and literature review, only three bisexual species of *Ottelia* are known from China. These are *O. guanyangensis*, a species described in 2018 (Li et al. 2018), *O. balansae*, and *O. alismoides*, the latter two recorded from “Flora of China”. Compared to the recorded three bisexual species, it was interesting that the population from Fengshan county had some unique flowers (e.g. white styles and over three flowers each spathe) and leaf traits (e.g. triplinerved with obvious cross-veins). We transplanted several individuals to the greenhouse at Wuhan Botanical Garden, Chinese Academy of Sciences, to observe the growth. Here we formally describe and discuss this taxon as a new species based on careful morphological observations and molecular phylogeny.

## Material and methods

### Morphological study

**Figure 2.**
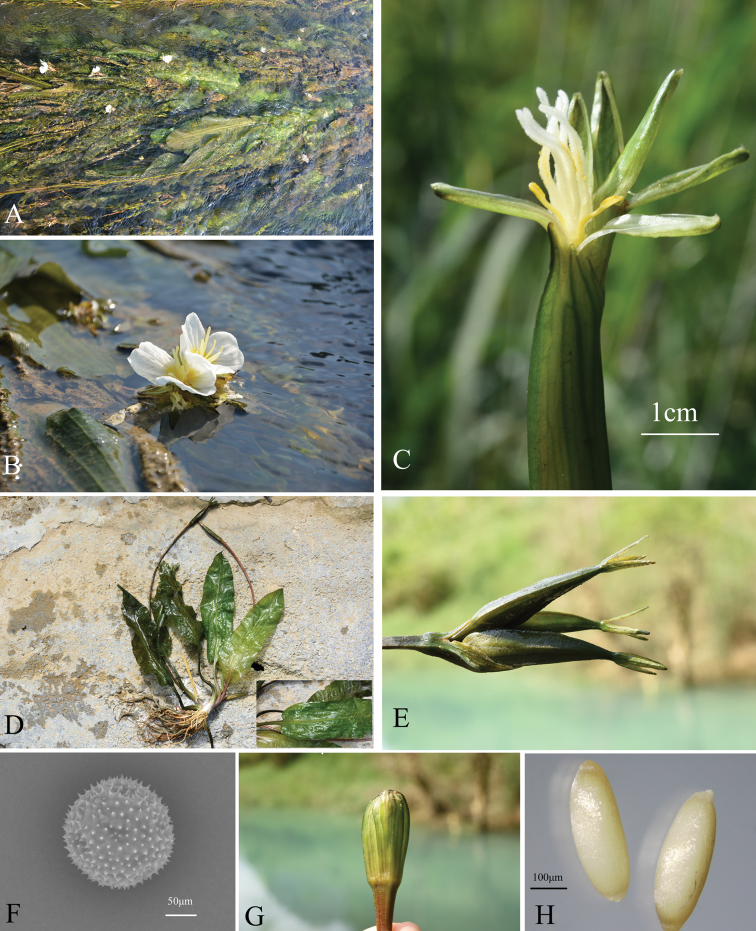
*Ottelia fengshanensis* Z.Z.Li, S.Wu & Q.F.Wang. **A** Habitat **B** flowering plant **C** bisexual flower with red-green sepals **D** individual and leaf: triplinerved with conspicuous cross veins **E** fruit: Hexagonal-cylindric with tiny wings **F** the character of pollens by SEM **G** spathe **H** seeds.

The morphological characteristics of the new species were collected during fieldwork in July 2018. We randomly selected 10 individuals, took pictures of each part and measured the characteristics of flowers, leaves and fruits (Fig. 2). The pollen grains of new species were gold-coated, and photographed using a Hitachi S-800 SEM system at Wuhan Botanical Garden, CAS. Simultaneously, we collected voucher specimens and several fresh leaves were dried using silica gel for DNA extraction. For further detailed morphological analysis, we transplanted five living individuals to a greenhouse at Wuhan Botanical Garden. We also observed the characteristics of flowers, leaves and fruits of these two bisexual species in our greenhouse for further comparative analysis (Table 1).

**Table 1.** The voucher information and GenBank accession numbers for the sequences of internal transcribed spacer (ITS) and three cp regions (*trn*S-*trn*G, *rbc*L and *trn*K5’ intron) in the present study.

Taxon	Individual code	Locality	Voucher no.	Acceession No.			
				ITS	*rbc*L	*trn*K5’ intron	*trn*S-*trn*G
O. acuminata var. jingxiensis	01	Jingxi, Guangxi	HIB-Otte010	MG751780	MH257624	MH257642	MH257660
	12	Debao, Guangxi	HIB-Otte009	MG751781	MH257628	MH257646	MH257664
	19	Du’an, Guangxi	HIB-Otte012	MG751782	MH257630	MH257648	MH257666
O. acuminata var. crispa	10_1, 10_2	Luguhu, Yunan	HIB-Otte011	MG751784/MG751785	MH257626/MH257627	MH257644/MH257645	MH257662/MH257663
O. acuminata var. acuminata	9	Heqing, Yunan	HIB-Otte003	MG751786	MH257625	MH257643	MH257661
	15	Jianchuan, Yunan	HIB-Otte006	MG751787	MH257637	MH257655	MH257673
	30	Caohai, Guizhou	HIB-Otte014	MG751788	MH257633	MH257651	MH257669
O. acuminata var. lunanensis	16	Shilin, Yunnan	HIB-Otte008	MG751789	MH257629	MH257647	MH257665
O. acuminata var. songmingensis	21_1, 21_2	Songming, Yunnan	HIB-Otte007	MG751790/MG751791	MH257631/MH257632	MH257649/MH257650	MH257667/MH257668
*O. balansae*	29	Huaxi,Guizhou	HIB-Otte005	MG751792	MH257634	MH257652	MH257670
*O. emersa*	41	Guigang, Guangxi	HIB-Otte004	MG751794	MH257638	MH257656	MH257674
*O. cordata*	40	Haikou, Hainan	HIB-Otte001	MG751795	MH257639	MH257657	MH257675
*O. alismoides*	42	Changping, Fujian	HIB-Otte002	MG751796	MH257640	MH257658	MH257676
*O. guanyangensis*	32	Guanyang, Guangxi	HIB-Otte015	MG751797	MH257635	MH257653	MH257671
	34	Guanyang, Guangxi	HIB-Otte016	MG751798	MH257636	MH257654	MH257672
*O. fengshanensis*	35	Fengshan, Guangxi	HIB-lzz51	MK531550	MK531552	MK531553	MK531551
*B. japonica*		Wuyishan, Fujian	HIB-Bly001	MG751799	MH257641	MH257659	MH257677

### Phylogenetic analysis

Total genomic DNA of one sample, collected from Fengshan county, Hechi city, Guangxi province, was extracted following Li et al. (2018). One nuclear DNA region (ITS) and three chloroplast DNA regions (*trn*S-*trn*G, *rbc*L and *trn*K5’ intron) were sequenced; the primers and PCR protocols followed Li et al. (2018). The same sequence regions from other species were downloaded from the NCBI (Table 2). The sequence alignments were made using MAFFT with default settings (Kuraku et al. 2013). The best nucleotide substitution model was detected using jModeltest 2.1.4 (Darriba et al. 2012) with the Akaike Information Criterion (AIC). The Maximum Likelihood (ML) analysis was made using IQtree with 5000 bootstrap replicates (Nguyen et al. 2003). The Bayesian Inference (BI) was analyzed by MrBayes v.3.2.6 (Ronquist and Huelsenbeck 2015), with 20,000,000 generations and four chains run with sampling after every 2000 generations. The first 25% of generations were discarded and a majority rule consensus tree (> 50%) was computed from the remaining trees. In order to test molecular support for species delimitation in *Ottelia*, the Poisson Tree Processes model for species delimitation (PTP) was applied to the tree with the following parameters: 500,000 generations; thinning: 100; burnin: 0.1 and seed: 123 (Zhang et al. 2013).

**Table 2.** Morphological characters comparison among *Ottelia fengshanensis*, *Ottelia guanyangensis* and *Ottelia balansae*.

Characters	*Ottelia fengshanensis*	*Ottelia guanyangensis*	*Ottelia balansae*
Flowers	bisexual	bisexual	bisexual
Sepals	1.0–1.5 cm, red green	1.0–1.5 cm long, red brown	2.0–2.5 cm long, green
Stamens	3;filaments 3.0–5.0 mm long	3; filaments 5.0–7.0 mm	3; filaments 4.0–5.0 mm
Ovary	5–10 cm long, hexagonal-cylindric to cylinder	4–5 cm long, hexagonal-cylindric	3.5–5.0 cm long, triangularcylindric
Styles	3, bifid to base,white	3, bifid nearly to base,yellow	3.5–5.0 cm long, yellow
Spathe	3-4 (3) flowered	2–5 flowered	3–11 flowered
Leaf shape	Linear or oblong, 30–70 × 8–14 cm,base rounded, apex acute or obtuse;petiole 8.0–10.0 cm long	linear, 15–50 × 2.5–4.0 cm, base rounded, apex acute, petiole 8.0–13.0 cm long	oblong or ovate, 20–40 × 6.0–8.0 cm, base truncate, rounded, or cordate, apex acute or rounded, petiole ca. 20 cm long
Texture	green, opaque, thick ca. 0.8 mm	dark green, opaque, thick ca. 1.2 mm	green, translucent, thick ca. 0.5 mm
Venation	trinerved with obvious cross veins, distance 4.0–6.0 cm to base, longitudinal veins 9	trinerved with obvious cross veins, distance 4.0–6.0 cm to base, longitudinal veins 9	basal veins, longitudinal veins 7
Fruit	hexagonal-cylindric, winged unobviously	hexagonal-cylindric, winged	narrowly elliptic, unwinged
Seed	fusiform, ca. 1.0 mm long	fusiform, ca. 1.5 mm long	cylindric to fusiform, ca. 3.0 mm long
Pollen	spheroidal, inaperturate, ca. 40 × 40 μm	spheroidal, inaperturate, ca. 35 × 45 μm	spheroidal, inaperturate, ca. 49 × 53 μm
Flowering time	April to November	April to October	June to November

## Results and discussion

The comparison among three bisexual species, *O. fengshanensis*, *O. guanyangensis* and *O. balansae*, is presented in Table 2. The new species had unique features, including the number of flowers, white styles, trinerved venation with distinct cross veins and longer leaf shape.

Morphological characters distinguish *O. fengshanensis* from the three bisexual species. For *O. alismoides*, there is only one flower in each spathe and it is easy to distinguish from the new species. However, *O. guanyangensis* and *O. balansae*, which are distributed in Guangxi province and Guizhou province respectively, are closest to the new species. The critical diagnostic characters of *O. fengshanensis* include having white styles, longer leaf shape and number of flowers in each spathe. Moreover, these three species are also isolated geographically, *O. fengshanensis* was only found in Fengshan county, but *O. guanyangensis* was found in Guilin city. *O. balansae* was only recorded in Guizhou province based on a recent survey. Karst terrain will play an important role in species divergence in this lineage.

Four sequence regions (ITS, *trn*S-*trn*G, *rbc*L and *trn*K5’ intron) were aligned and concatenated into a 3623 bp sequence. 605 variable nucleotides were detected. Two clades were displayed with high support (BS= 70, PP= 0.7). PTP analysis further recognized four species with *O. fengshanensis* having the highest support (0.678). Based on phylogenetic analyses, *O. fengshanensis* was resolved as sister to *O. guanyangensis* with high support (BS= 100, PP= 1.0) and only distantly related to *O. balansae*, which clusters together with *O. acuminata* (Fig. 4), and based on PTP analysis, *O. balansae* was not supported as a species, but was more likely to be treated as a bisexual variety of *O. acuminata*. In combination, the morphological and molecular phylogenetic analyses support that *O. fengshanensis* is a distinct species closely related to *O. guanyangensis*, a species also distributed in Guangxi province.

*Ottelia* possesses complex floral traits and may have bisexual and unisexual flowers. Based on the previous studies (He 1991, Chen et al. 2012) bisexual flowers have evolved multiple times in *Ottelia*. Here we report a new bisexual species *O. fengshanensis* and verify that bisexual flower indeed has multiple origins in *Ottelia*. *Ottelia fengshanensis* probably has a common ancestor with the unisexual O. acuminata var. songmingensis. Besides, we also suggest that *O. balansae* should be treated as a variety of *O. acuminata*. This point has also been put forward by Yu Ito et al. (2019). It will also help us have a better understanding of the diversity and evolution of sex evolution in *Ottelia*.

### Description of the new species

#### Ottelia fengshanensis

Taxon classificationPlantaeAlismatalesHydrocharitaceae

Z.Z.Li, S.Wu & Q.F.Wang sp. nov.

559D0913-8825-5336-83BB-9B591BEF2421

urn:lsid:ipni.org:names:77202741-1

Fig. 3

##### Description.

The new species is similar to *Ottelia guanyangensis* in having bisexual flowers, three stamens, but differs through having (3)-4 flowers in each spathe (vs. 2–5), white styles (vs. yellow), green leaves (vs. dark green) and by fruits which are tiny winged (vs. obviously winged).

##### Type.

CHINA. Guangxi, Hechi City, Fengshan County, elev. 507 m, 24°34'20"N, 107°10'17"E 11 September 2018, *Z. Z. Li & S. Wu-Otte51* (holotype HIB-lzz51!).

Annual or perennial herb. Rhizome, short. Leaves entirely submerged, dark green and opaque, linear or oblong, 30–70 × 8–14 cm, base rounded, apex acute or obtuse; longitudinal veins 9; midrib conspicuous, stretched to the apex, becoming trinerved with obvious cross-veins at a distance of 5–7 cm from the base; petiole smooth, green, 8.0–10.0 cm long, the base expanded into a sheath. Spathe oblate, ca 3. 0 × ca. 3.5 cm, warty along edges or smooth, longitudinally ribbed and winged on the lateral margins, containing 3–4 (3) flowers; flowers bisexual; sepals reddish green, 1.0–1.5 × ca. 0.5 cm, with longitudinal ribs; petals white with yellow base, obovate, ca. 2.0 × ca. 2.0–2.5 cm, with longitudinal pleats; stamens 3, opposite to sepals, anthers elliptic, connective obscure, filaments 3.0–5.0 mm long; glands 3, 0.5–1.0 × 0.5–1.5 mm, opposite to petals, pale yellow. Ovary hexagonal-cylindric to the cylinder, 5–10 cm long, with 3 carpels; styles 3, white, slender and hairy, 1.2–1.5 cm long, stigma bifid, divided to base; stigmas 6, liner and hairy, ca. 8 mm long. Fruit a hexagonal-cylindric capsule, with 6 inconspicuous wings, dark green, with persistent calyx, 4.0–9.0 cm × ca. 6.5 mm, always longer than spathe. Seeds numerous, fusiform, ca. 1.0 mm long, both ends hairy. Pollen, subglobose, ca. 40μm in diam, with spiny granules.

**Figure 3.**
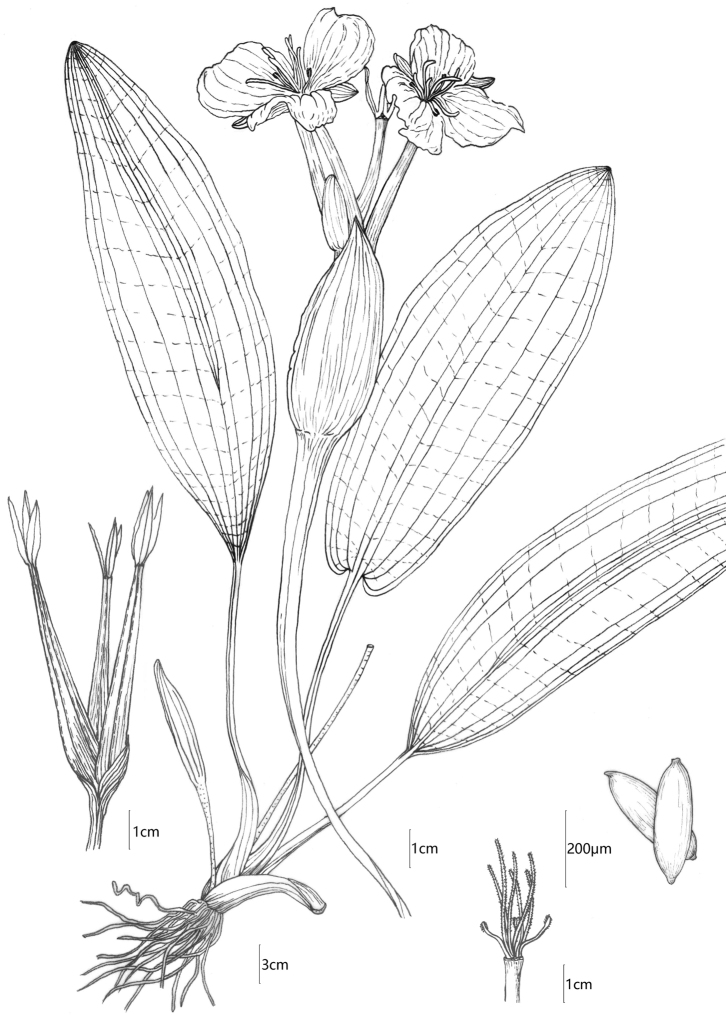
Illustration of *Ottelia fengshanensis* Z.Z.Li, S.Wu & Q.F.Wang. Drawn by Shuai-Jie Li.

**Figure 4.**
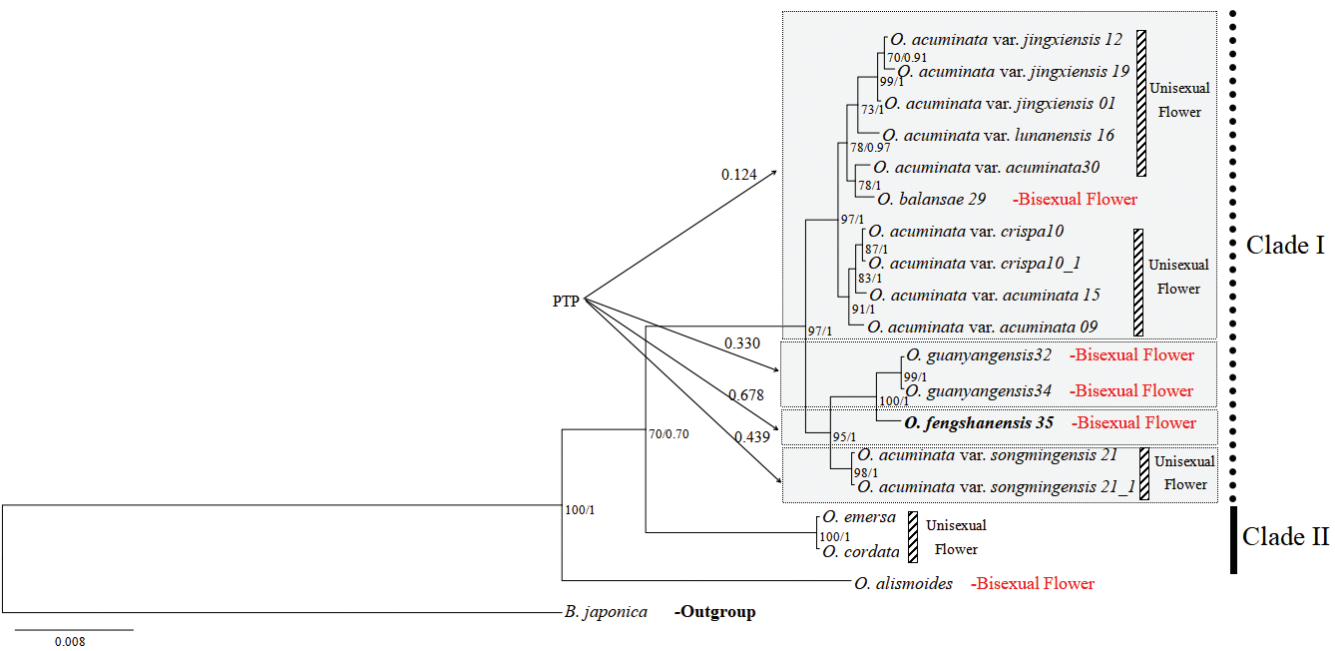
Phylogenetic tree and PTP analysis of *O. fengshanensis* sp. nov. and *Blyxa japonica* as an outgroup. Posterior probabilities (PP > 0.70) and bootstrap values (BS > 70) based on Bayesian Inference and maximum likelihood (ML) analysis are shown above the branches.

##### Distribution and habitat.

*Ottelia fengshanensis* is known from a single population in Fengshan County, Guangxi Province, China. The species inhabits a karst river less than 1.5 m in depth. Due to the complex underground river system in the karst region, it is probable that the species occurs in nearby areas as well.

##### Conservation status.

Only one population of new species was found at Fengshan County, Guangxi Province, China. Although it might be distributed in adjacent karst rivers. Until now, approximately 50–100 individuals were found in a single population. However, there is not enough information on population size and dynamics. According to the IUCN Red List Categories and Criteria (IUCN 2017), we suggested that the species be evaluated as Data Deficient (DD).

##### Phenology.

The new species was found in flower from April to November.

##### Etymology.

The epithet is derived from the name of Fengshan County, which is the only known locality of occurrence.

##### Other specimens examined (paratypes).

CHINA. Guangxi, Hechi City, Fengshan County, elev. 507 m, 11 September 2018, *Z. Z. Li & S. Wu Otte 056* (HIB!)

## Supplementary Material

XML Treatment for Ottelia fengshanensis
